# Integrating frontiers: a holistic, quantum and evolutionary approach to conquering cancer through systems biology and multidisciplinary synergy

**DOI:** 10.3389/fonc.2024.1419599

**Published:** 2024-08-19

**Authors:** Matheus Correia Casotti, Débora Dummer Meira, Aléxia Stefani Siqueira Zetum, Camilly Victória Campanharo, Danielle Ribeiro Campos da Silva, Giulia Maria Giacinti, Iris Moreira da Silva, João Augusto Diniz Moura, Karen Ruth Michio Barbosa, Lorena Souza Castro Altoé, Lorena Souza Rittberg Mauricio, Luíza Santa Brígida de Barros Góes, Lyvia Neves Rebello Alves, Sarah Sophia Guedes Linhares, Vinícius do Prado Ventorim, Yasmin Moreto Guaitolini, Eldamária de Vargas Wolfgramm dos Santos, Flavia Imbroisi Valle Errera, Sonia Groisman, Elizeu Fagundes de Carvalho, Flavia de Paula, Marcelo Victor Pires de Sousa, Pierre Basílio Almeida Fechine, Iuri Drumond Louro

**Affiliations:** ^1^ Núcleo de Genética Humana e Molecular, Universidade Federal do Espírito Santo (UFES), Vitória, ES, Brazil; ^2^ Laboratório de Oncologia Clínica e Experimental, Universidade Federal do Espírito Santo (UFES), Vitória, ES, Brazil; ^3^ Grupo de Bioanalítica, Microfabricação e Separações (BioMicS), Universidade de São Paulo (USP), São Carlos, SP, Brazil; ^4^ Laboratório de Estudos Neuroquímicos, Universidade Federal do Rio Grande do Norte (UFRN), Natal, RN, Brazil; ^5^ Instituto de Biologia Roberto Alcântara Gomes (IBRAG), Universidade do Estado do Rio de Janeiro (UERJ), Rio de Janeiro, RJ, Brazil; ^6^ Bright Photomedicine, Tergos Pesquisa E Ensino S.A., São Paulo, SP, Brazil; ^7^ Group of Chemistry of Advanced Materials (GQMat), Department of Analytical Chemistry and Physical-Chemistry, Federal University of Ceará (UFC), Fortaleza, CE, Brazil

**Keywords:** neoplasms, carcinogenesis, biological evolution, chaos and quantum theory, dynamics, translational science

## Abstract

Cancer therapy is facing increasingly significant challenges, marked by a wide range of techniques and research efforts centered around somatic mutations, precision oncology, and the vast amount of big data. Despite this abundance of information, the quest to cure cancer often seems more elusive, with the “war on cancer” yet to deliver a definitive victory. A particularly pressing issue is the development of tumor treatment resistance, highlighting the urgent need for innovative approaches. Evolutionary, Quantum Biology and System Biology offer a promising framework for advancing experimental cancer research. By integrating theoretical studies, translational methods, and flexible multidisciplinary clinical research, there’s potential to enhance current treatment strategies and improve outcomes for cancer patients. Establishing stronger links between evolutionary, quantum, entropy and chaos principles and oncology could lead to more effective treatments that leverage an understanding of the tumor’s evolutionary dynamics, paving the way for novel methods to control and mitigate cancer. Achieving these objectives necessitates a commitment to multidisciplinary and interprofessional collaboration at the heart of both research and clinical endeavors in oncology. This entails dismantling silos between disciplines, encouraging open communication and data sharing, and integrating diverse viewpoints and expertise from the outset of research projects. Being receptive to new scientific discoveries and responsive to how patients react to treatments is also crucial. Such strategies are key to keeping the field of oncology at the forefront of effective cancer management, ensuring patients receive the most personalized and effective care. Ultimately, this approach aims to push the boundaries of cancer understanding, treating it as a manageable chronic condition, aiming to extend life expectancy and enhance patient quality of life.

## 1 Introduction

Cancer occurs when cells stop working for the collective and become selfish. These changes transform the cells, giving them competitive advantages. Cancer manifestations are similar to the biological processes observed in unicellular organisms. Oncogenes versions are present in viruses, unicellular organisms, and invertebrates (1, 2). Nowell (3) made comparisons between the selective forces that influence cancer cells in the human body with those affecting individuals within populations in nature. Their increasing genetic instability contributes to elevated genetic diversity within the cancer cell population and is likely to amplify phenotypic diversity (4).

The basis for the somatic mutation theory (SMT) hypothesis originates in 1914, when Theodor Boveri postulated that a combination of chromosomal and mutagenic defects could result in cancer. Thus, somatic mutation was structured as a causal event for the onset of cancer. Consequently, SMT was extrapolated to all tumors and formalized a 100-year history, with an inability to show appreciable clinical benefits for all types of tumors (2). This is because the reductionism regarding the unique importance of genes and the inflexibility towards new developments in the field of oncology “fabricated” a research area with incorrect interpretations of data, as it stood out as the most parsimonious explanation. As a result, new scientific approaches will be required to integrate, explain, and direct effective treatments against cancer (2).

## 2 Brief interpretation of cancer

Proceeding with such aforementioned facts, it is noted that cancer life cycle follows the life cycle of common ancestors such as amoebozoans, metazoans, and fungi (AMF), which are governed by the Evolutionary Biology of Cancer Cells (ECCB) Theory. This is because genes, genetic modules, and gene regulatory networks of pre-metazoic cellular systems may have been preserved in the ancestral genome of metazoans and humans. Furthermore, genomic integrity can be restored through homotypic cells and nuclear fusion, resulting in the formation of high-degree polyploids known as multinucleated genome repair syncytia, or by hyperpolyploidization (5–7).

Going deeper genetically, cumulative somatic mutations over the evolution of a cancer cell shape its genome, and a portion of this trajectory can be reconstructed through the analysis of whole genome sequencing data (8–10). Moreover, the nearly universal presence of cancer indicates the roots of its evolutionary history. For example, the existence of tumors in dinosaurs has been recorded on several occasions (11). According to Weinberg (12), ancestral forms of oncogenes were already manifesting among primitive metazoans, which constitute the common ancestral lineage of both chordates and arthropods. Other more recent genetic research conducted on freshwater Hydras suggests that the *MYC* oncogene, specific to humans, has an evolutionary origin that can be traced back at least 600 million years ago (13).

Nevertheless, the continuous complex adaptation of cancer is regulated by nonlinear feedback systems between genetic instabilities, environmental signals, cellular protein flows, and gene regulatory networks (14, 15). Tumor genomes are subject to slow microevolutionary and punctual macroevolutionary changes, according to the 2nd law of thermodynamics by Boltzmann, Darwin’s selection principle, and entropy causing mutations that lead to increased genetic variation, promoting the development of cancer and its phylogenetic evolution (14, 15).

Despite so many advances, cancer still stands out as a set of challenging diseases, but why? Why, even with the establishment of the “national war on cancer” by President Nixon, has a cure not been achieved? How does precision oncology address these issues? Is it a grand illusion? Does research based on large datasets provide solid scientific foundations or simply a “straw set”? Numerous other questions arise throughout the long journey of oncology. Multidisciplinary, translational, and holistic approaches are crucial for elucidating and effectively implementing adaptive therapeutic strategies in oncology (16–18).

In addition to restructuring the approach to cancer, new understandings of how to tame or control cancer need to be incorporated, avoiding reliance on ineffective “death strategies” that induce aggressive tumor adaptations. A deep understanding of evolutionary, holistic, and translational parameters, along with chemical and physical principles, is expected to usher a new era of more personalized, single-cell-based oncology capable of encompassing new adaptive therapies for the improvement of patient survival and quality of life (19).

Despite technological advances and research, the difficulty in winning the battle against cancer goes through various spheres. The mysticism surrounding the mechanisms that promote the emergence of tumors, the heterogeneity of tumors, the lack of standardization to responses to drugs and treatments, and issues inherent to tumor progression are some of the key points. Cancer cells are capable of accessing stages of evolutionary progression that corroborate their unstable character and physiological plasticity (20). Such configurations explain why cancer is today one of the most complex and challenging diseases, a product of both evolutionary biology, genetics, epigenetics variability and quantum dysfunctions.

## 3 Brief interpretation of cancer therapy

Cancer demands new therapeutic paths that are more integrated, multidisciplinary, and translational, cultivating an intuition for systems oncology and connected with other challenging areas such as regenerative biology, biotechnology, bioinformatics, systems biology, among others, as reviewed by Alves et al. (21), Casotti et al. (22), Casotti et al. (23) and Meira et al. (24). Thus, by tracing the evolutionary history of cancer, the influences of cellular biological variations, such as aneuploidy and polyploidy, will be understood. Structures like syncytia and cell fusion phenomena reveal the complexity of cellular interactions that lead to malignant transformation.

Moreover, the “dive” into the genomic and epigenomic chaos illustrates how it contributes to oncogenesis. New hypothesis and creative insights are demanded, such as incorporation of the chaos theory and quantum principles to understand the nonlinear dynamics of cancer, and to propose non-conventional treatments and researches. Translational and adaptive research, single-cell analysis technologies, systems biology, comparative oncology, quantum biology, and chaos theory will provide unprecedented elucidation about cancer evolution.

The fight against cancer faces significant hurdles, including the challenge of overcoming treatment-induced resistance and the absence of a definitive cure. The integration of evolutionary and systems biology into oncological research holds promise for developing adaptive, effective treatments by leveraging comprehensive data and multidisciplinary approaches, potentially offering new strategies to control and mitigate cancer. The current era in cancer research is a pivotal moment, poised for transformative advancements through technological and interdisciplinary efforts. Achieving this vision requires breaking down disciplinary barriers, embracing flexibility in scientific innovation, and fostering open communication to enhance patient care and quality of life, setting a course towards managing cancer more effectively and sustainably. By undertaking such a comprehensive and innovative exploration of the intersections between multiple complex areas, the intent is to answer the following guiding question: How do the mathematical, chemical, physical, and biological characteristics and peculiarities of cancer highlight the need for a translational, holistic, single-cell, adaptive, and evolutionary approach? As a result, it will be possible to highlight the inherent challenge in studying cancer through crosstalk of theories, experimental analyses, and heterogeneous data, to innovate through pioneering in diagnosis, monitoring, and treatment, and to reach numerous promises and challenges in the search for more effective and personalized therapies.

### 3.1 Evolutionary biology and cancer

The great cellular anarchy within the microcosm of a multicellular organism that provides tumor origin has its roots extending through the vast domain of life (25–31). Thus, embarking on the journey through the phylogenetic history of cancer, interpreting the evolution of cells traces an interpretation of cancer to its most primitive origins (31). Protists, fungi, invertebrates, and vertebrates, each evolutionary group added layers of complexity that paradoxically facilitated cancer emergence. In multicellular organisms, the evolution of specialized cell types under strict genetic and epigenetic control became a strategy to harmonize cell proliferation and differentiation, a delicate balance often subverted in cancer (25–31).

Tumorigenic evolutionary process in miniature, where cells within a tumor compete, evolve, and adapt, integrates with a diversity of cell types, and there is a prominent contribution of asymmetric cell division to phenotypic heterogeneity (32). Such processes are modulated by continuous interaction with the tumor microenvironment, highlighting the co-evolution of tumor cells and their niche (33).

Genomic studies support the idea that the cancer genome evolved hundreds of millions of years ago, long before the emergence of multicellular organisms, as emphasized by biology (ECCB) (34, 35). Thus, within an eco-evolutionary dynamic, there is an overlap between genes and pathways related to metazoans, fungi, and amoebas in cancer, highlighting regulatory capabilities for both the establishment and reversal of cellular fate and tissue integrity in the tumor mass (36).

Among current comparisons supporting these relationships, the social selection observed in slime molds, organisms that transition between uni- and multicellularity, stands out. They represent the importance of spatial and pre-adaptive aspects as promoters of evolution under selection in the social context, reinforcing multicellular collective behavior, similar to tumors (37). In summary, fungi, with their dynamic and plastic genomes responding to environmental stress, present increased rates and numbers of adaptive routes arising from aneuploidy, resembling cancer cells chaos. This makes fungi a good model for combating adaptation in tumors through “evolutionary traps” (38).

Additionally, shared genomic instability between fungi and tumors reveals special pathways for the formation of multinucleated cells (polyploidization), syncytia, fusion of mononuclear cells, and cell-in-cell structures, as dominant survival strategies supporting biodiversity in invertebrates and protists (39). This likely plays an analogous role in cancer cells. Building on these claims, cancer expresses a cellular system capable of switching between multicellular and unicellular subsystems with “cellular regression” involving unbalanced energetic sets (40).

Regarding this polyploidization, cancer progresses as a “runaway locomotive” reflecting genomic instability promoting cellular diversity and acquisition of new capabilities, such as drug resistance and metastatic potential (41–43). Thus, there is a dynamic, probabilistic, and unpredictable nature that pervades cellular resilience and adaptation to the energetic turbulence underlying cellular dynamics in the tumor, serving as a promising source for new innovative therapeutic approaches (44–46).

#### 3.1.1 Process resulting from evolution

**Figure 1:**
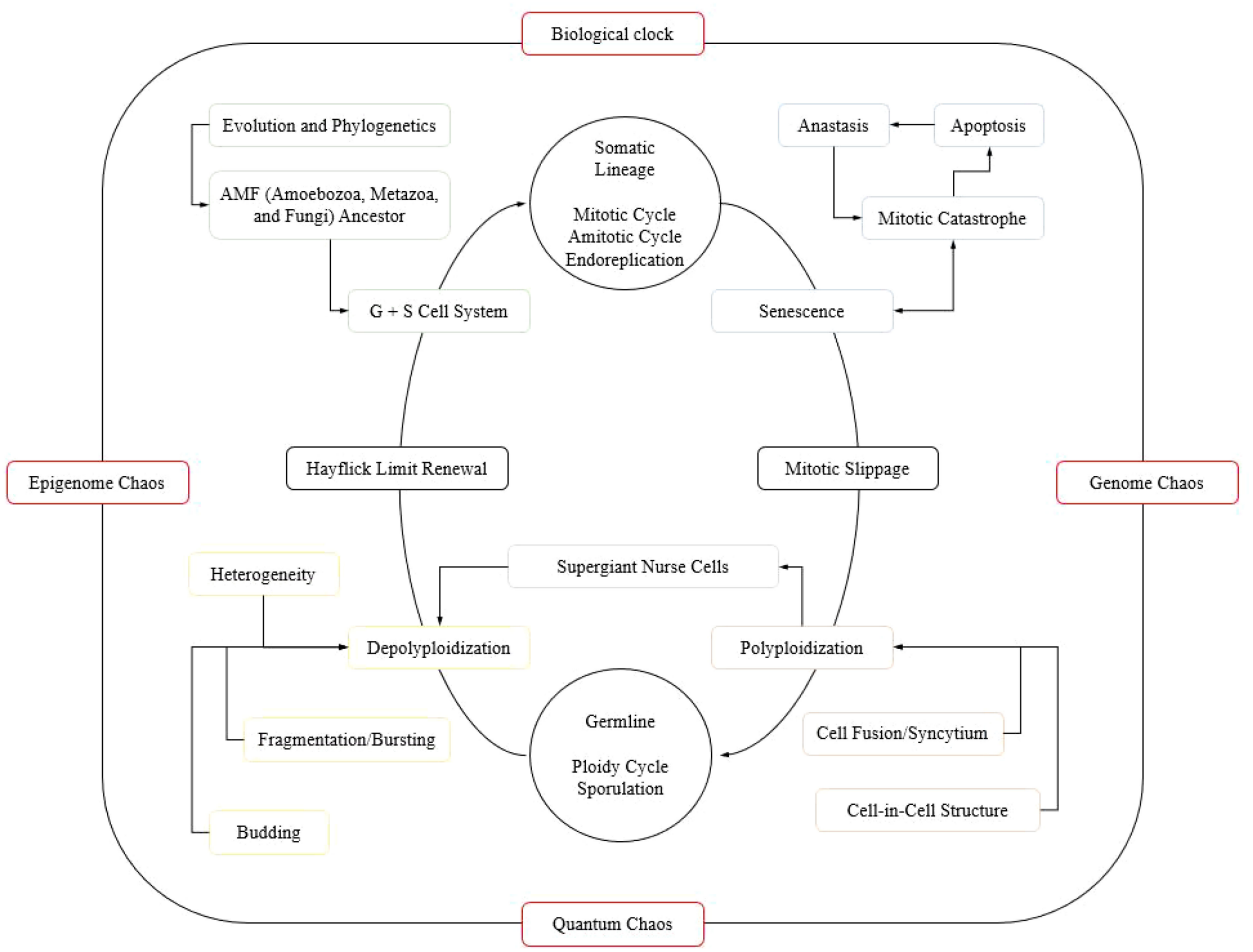
Theoretical compilation on tumor evolution. Based on bibliographic data and isolated schemes, according to Heng and Heng (16), Casotti et al. (19), Niculescu (34), Niculescu (35), Erenpreisa et al. (48), Uthamacumaran (49), and Russo et al. (50). Cancer evolution involves a somatic lineage characteristic, immersed in proliferation following mitotic, amitotic and endoreplication cycles. However, this process reaches a cellular limit that leads to senescence which can bridge to a mitotic catastrophe followed by apoptosis with or without anastasis, but with a connection to mitotic slippage capable of guiding polyploidization. Polyploidization seems to act as a central connective hub for cell fusion mechanisms, syncytium formation, and cell-in-cell structures. Through this, a germline phase prone to the perpetuation of genomic, epigenomic, and quantum chaos for descendants is reached through depolyploidization. What might be imagined to be entirely random, a system dynamism through continuous self-organization also provides resistance to extinction. And have a phylogenetic regression to unicellular traits under an evolutionary access to ancient regulatory genetic networks and transcriptional regulatory complexes capable of providing singularities, such as bursting/fragmentation, budding mechanisms and formation of supergiant nurse cells, promoting cellular diversity according to intra- and intertumoral heterogeneity, breaking the barriers of the Hayflick limit, which is sustained by a common G + S ancestral cellular system from amoebozoans, metazoans, and fungi (AMF). From this perspective, cancer modulation is present through a mechanistic compilation of balanced processes on a profound systemic dynamism of genome, epigenome, and quantum chaos, and regulation of the biological, physiological, pathological, and normal circadian clock, requiring a robust therapeutic approach through lenses of personalized and adaptive approaches for effective modulation and mastery of cancer cells.

Throughout the “tree of life,” cancer can be interpreted as a cheat against a complex cooperative multicellular system, as evidenced by cancer hallmarks, demonstrating selfish characteristics of proliferation, perpetuation, and survival against extinction, similar to various unicellular and multicellular organisms (19, 47). In this cancer evolution process, cells proliferate through various cycles, ultimately hitting a limit that leads to senescence or cell death, but also can lead to polyploidization—where cells gain extra sets of chromosomes. This polyploidization connects to various mechanisms that promote cell diversity and can bypass cellular aging limits. It serves as a gateway to genetic chaos but also to self-organization and survival strategies, reflecting ancient cellular traits. This complex process underscores cancer’s adaptability and the need for personalized, adaptive therapeutic strategies to effectively manage and control cancer progression, for further clarification on the relationship between evolution, cellular variability, and cancer, **Figure 1** is highlighted.

### 3.2 Systems biology, quantum, chaos and cancer

The dynamic complexity of cancer underscores the demand for principles from systems biology and chaos theory, making it crucial to consider the tumor system as a whole, including its dynamic interactions with the host (51). From this perspective, understanding cellular signaling networks and metabolic pathways in their entirety becomes a strategy to identify critical points in regulation that can be targeted for therapeutic interventions (52, 53). Moreover, by incorporating the understanding of tumor responses to cell stresses, such as polyploidy and syncytium, the layer of cellular plasticity and adaptability is added according to the principles of Mendelian or non-Mendelian, as McClintock’s inheritance (54–56).

In line with the various relationships existing in a tumorigenic mass, the application of quantum physics to biology and medicine can reveal underlying mechanisms of these diseases, challenging existing paradigms, proposing new approaches for treatment and diagnosis, while also elucidating fundamental biological processes (57, 58). Among directly applicable examples, it’s worth highlighting: i) the butterfly effect (the idea that small variations in biological systems can lead to large effects) by emphasizing the difficulty of predicting cancer, requiring a deeper understanding of nonlinear and chaotic dynamics (59); and ii) synchronicity and coherence as critical phenomena for maintaining homeostasis and normal cell functioning, which are lost as cellular and molecular self-organization occurs in the tumor (60, 61).

#### 3.2.1 Walking through complexity

Based on these initial highlights, it is emphasized that a cancer’s complexity and chaos act together in a system of stability and instability, with multiple stages that can be mathematically described by the chaos theory and visualized in fractal geometry (62). In other words, it is a set of diseases characterized by unpredictability and complexity. Tumor evolution is immersed in an environment outside thermodynamic equilibrium, essential for inducing self-organized complexity cancer as the source new structures, survival strategies, tumor microenvironment adaptation, genomic reorganization, global stability/local instability, and activation of a pathological biological clock (16, 19, 20, 48–50, 63).

System chaos can cause a “creative disorder,” by a two-phase evolution of connecting contributions of karyotypes and genes: chaos induced by stress (providing macroevolutionary genomic shuffling) and adaptation (16, 19, 48). Moreover, the quantum adaptive evolution (= quantum states, i.e., qubits) highlights tumor evolution as a co-dependent microenvironment selection. Cancer cells have an environmental dominance that provides selective adaptation of epigenetic landscapes in favor of survival phenotypes (49). Simultaneously, changes in epigenetic traits impact on epigenome chaos needed for inter- and intratumoral heterogeneity, involving disordered hyper- and hypomethylation in various DNA segments (50).

Tumor is as a system far beyond the SMT, presenting shared evolution among multiple unicellular and multicellular organisms, showing adaptive genetic regulatory networks on the brink of order and chaos. Cell fate can be modulated by oscillations between cellular senescence and reprogramming self-organization, related to unicellularity, an existing phylogenetic feature, and negative selection of variants unsuitable for survival (48).

A key matter about evolutionary cancer concepts is how to provide more effective treatments based on the cancer’s evolutionary progression, allowing tumor control and modulation. Evolutionary comparative oncology supports studies of tumor cheating, risk stratification and clinical management, helping cancer prevention research programs based on detection and suppression (47). Additionally, translate into better outcomes and positive treatment responses (64).

Owing to the multifactorial nature of oncogenesis, attacking all possible molecular agents simultaneously is currently, and impractical due to a lack of solid investigative foundations (16). Therefore, holistic studies with better experimental designs, fewer misleading preclinical trials, and vague interpretations are needed (65), along with new approaches based on analytical systems to decipher epigenome, genome and quantum chaos (50), understanding of “evolutionary traps” by comparative oncology (38), and the inclusion of adaptive therapy for clinical protocols. This therapy is a promising therapeutic strategy, considering the evolution by a temporal and spatial microenvironment variability, as well as disturbances induced by therapy, providing a stable tumor burden with more chemosensitive cells (64). For such development, we need to translate between basic and clinical research, incorporating multidisciplinary teams, interorganizational collaborative models, sustainable market competition, and formalizing more interinstitutional partnerships (66).

#### 3.2.2 News due to holistic initiatives

As a result of numerous prominent studies on integrated areas aimed at elucidating new paths at the forefront of knowledge being explored in oncology, the increasingly complex functioning of cancer genome, controlled at different levels (1- DNA base sequence with inherited information, 2- Epigenetic pathways with protein interactions and feedback cycles, and 3- Genome architecture and organization, activating or suppressing interactions), is also influenced by environmental stress, which is immersed in coordinated biological, chemical, and physical changes, based on self-organized complexity (67, 68). Such changes in cellular activities and cell fate are associated with phase transitions of a cooperative “control architecture” based on networks and a “nucleotype” under coarse control, as with polyploidy (67, 68).

Despite intense complexity, cancer cells undergo causal effects and feedback that link the dynamics of heterogeneous and evolving tumors with changes in metabolic, inflammatory, nutrient competition, and immune response, giving rise to the adaptive complexity of cancer (69). Thus, a possible modulation by the immune system can restore immunosurveillance and avoid immune editing and escape (69). Other modulations may be related to the diversity of large-scale cross-genomic data sets, which, when combined with new evolutionary approaches, allows predicting the temporal order of somatic events that arise during tumorigenesis (70). In addition, laws and physical principles and the “field effect” that define the behavior of matter add foundations regarding tumor heterogeneity, drug resistance, and the ecological behavior of multiple cells with reproductive fitness and degrees of sensitivity to drugs, along with new therapeutic delivery strategies, from mechanics to evolution and from chemistry to nanotechnology (70). Furthermore, quantum physics displays principles that can act in the clinical area (70, 71).

Just as more robust modulations exist, there are subtle controls immersed in a tangle of evolutionary insights about the macro and microheterogeneity acting on the evolutionary plasticity of cancer (72). Recent technologies for single-cell sequencing and tracking provide an expansion of angles in more effective therapeutic intervention, allowing the delineation of spatial structures of subclonal architectures, detecting, tracking, and treating clinically dominant subclones (subjected to drugs, doses, and variable time under changes in the cell cycle and cell fate) in a live cell model, quantifying the generation of *de novo* mutations, building genotype-phenotype maps, and mapping dynamic fitness landscapes (72–74).

### 3.3 Translational and adaptive research in oncology

Translational research in oncology represents a critical bridge between scientific discoveries of basic research and clinical applications because increasingly molecular tools are added to sophisticate clinical aspects. Translational studies have been improving preclinical models applicable to therapeutic development, thereby advancing both diagnosis and therapy through molecular characterization of human tumors and continuous adaptation of therapeutic approaches to tumor characteristics evolution (75, 76). Thus, new paradigms and initiatives emerge.

The comparative oncology area regains significant attention in the oncology field, especially with innovative trends of One Health and the search for new models that can either replace classic model organisms or complement the mechanistic elucidation of cancer. Comparative oncology offers insights into why some species vary in their susceptibility to cancer and the mechanisms responsible for the diversity of cancer defenses (77). Additionally, clinical trials in veterinary cancer patients provide an opportunity to evaluate new therapeutics in a setting that recapitulates many of the key features of human tumors, improving cost-effectiveness and protocol efficiency for drug discovery, as well as incorporating new One Health perspectives into comparative and translational medicine (78, 79).

In addition to advances in comparative oncology, significant breakthroughs are added by single-cell biology studies, as it becomes possible to perform specific mapping of each tumor cell, highlighting intratumoral heterogeneity. Applications illustrate the power of this new area, such as the construction of single-cell atlases that unveil the evolution of cell types across Metazoa, allowing the identification of unique cellular states and conserved evolutionary traits, providing valuable insights into the cellular mechanisms underlying cancer (80). Single-cell tracking through microscopy or sequencing also elucidates real-time cellular dynamics, such as single-cell transcriptomics detailing dynamic mechanisms of cell fate decisions, crucial for identifying cancer precursor cells and therapeutic targets (81).

#### 3.3.1 Brief positive outcomes

Personalized therapeutic approaches as well as adaptive therapy based on real-time observation and response to disease progression stand out as a personalized, evolutionary, and dynamic approach to cancer treatment. This therapy incorporates an evolutionary and ecological model that allows continuous treatment adjustments based on patient response, aiming to apply a stable tumor burden, allowing the survival of chemosensitive cells and suppressing the proliferation of chemoresistant subpopulations, enhancing therapeutic efficacy and minimizing toxicity (64). In summary, the conceptual advancement of adaptive therapy has been adding new methodologies regarding multimodal approaches and also the discovery of new non-invasive imaging biomarkers that can be evaluated with PET/CT (Positron Emission Tomography and Computed Tomography Scans) or MRI (Magnetic Resonance Imaging) images and advanced single-cell tracking microscopy (82, 83).

**Figure 2:**
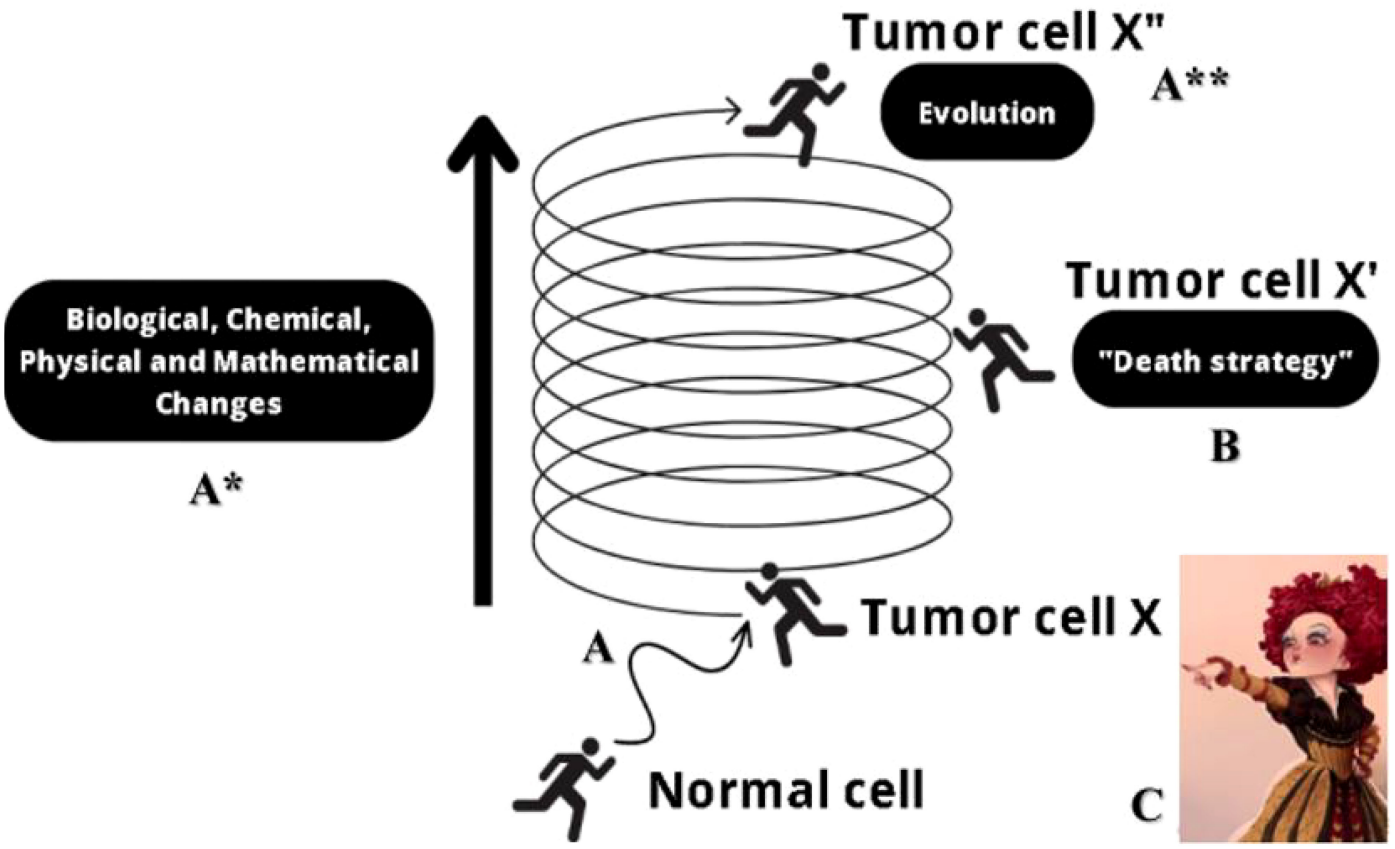
Multidisciplinary dialogues involve a prominent research path capable of controlling or “taming” the tumor cell. Throughout the vital journey of cells, they constantly encounter environmental fluctuations and adapt in response to these changes, following a path full of dynamism **(A)**. However, the emergence of an imbalance in the ability to correct damage and maintain harmony between adjustments and fluctuations can trigger the path to carcinogenesis (tumor cell X). Thus, in the incessant cycle of chaotic multiplication characteristic of cancer, a continuous change in the biological, physical, chemical, and mathematical aspects is observed with each new tumoral generation (A*). Simultaneously, the complexity of the expanding tumor emerges as a significant challenge for the survival of the host organism, and, to face this challenge, various therapeutic strategies adopt a “death strategy” view (that is, an aggressive approach of intense attack on the tumor, an adversary to be exterminated), offering cancer new evolutionary opportunities. This is because, by opting for aggressive treatments as a routine instead of promoting the death of tumor cells, beneficial evolution can be induced for resistant and competitive cells, which accompany an “arms race” as explicit in the Red Queen hypothesis (**B** -tumor cell X’-, **C**). Thus, the outcome is an advanced tumor cell, accumulating changes that allow it to oscillate between different attractor states and interact with various genetic networks, culminating in devastating clinical outcomes, such as resistance, relapse, dormancy, metastasis, anastasis, and others (A**, tumor cell X”). Source: Adapted from Casotti et al. (30). Create with Biorender.

Finally, experimental and clinical research synergistically connected to mathematical modeling and computational simulations promoting the integration of components and variables faithful to the reality of carcinogenesis, projection, and validation of therapeutic protocols, and translation according to adaptive methodologies, are joined by a multidisciplinary scientific approach integrated with dynamic programming and biophysical studies in the identification of quantum mechanical processes, supplementing innovations to enhance the ability to “educate” or control or “tame” the tumor (84–86). Multidisciplinary research emphasizes controlling cancer by understanding its adaptability to environmental changes and the risks of aggressive treatments, which may inadvertently promote resistant cancer forms. This approach advocates for a blend of comparative, translational, and holistic oncology, along with single-cell biology, to effectively tackle the complexity of cancer evolution and treatment resistance, stressing the need for adaptable and nuanced therapeutic strategies and, for further clarification on the relationship between translational, adaptive research and cancer, along with multidisciplinary principles, a summary figure of main terms and concepts guiding such discussion were addressed, see **Figure 2**. Significant progress in cancer biology research necessitates a more inventive and intelligent approach, one that harnesses the power of single-cell biology along with a systems or holistic view of biology. Moreover, staying adaptable to ongoing innovations is crucial for maintaining equilibrium in this challenging field, serving as a foundational pillar for comparative and translational oncology.

### 3.4 Perspective

This article highlights the broad spectrum of opportunities in oncology that comes from merging genetics insights, bioinformatics, quantum biology, evolutionary biology, systems biology, single-cell biology, translational research, and additional relevant fields. These areas, when combined with teamwork across professions and the embrace of cutting-edge technologies, show great promise in advancing the ways we diagnose, comprehend, and treat cancer. A comprehensive and interdisciplinary strategy, emphasizing energetic interventions, molecular coherence, dynamics of cell migration, among other techniques, is crucial for addressing the complexity of cancer. Such an approach paves the way for more efficient and tailored treatments, underscoring the critical role of innovative practices in modern medicine.

The intersection between quantum biology and modern medicine, as discussed by Restian (87) and Waring (88), reinforces the idea that cancer is a complex system that requires an integrative approach for its understanding and treatment. Carcinogenesis is addressed from multiple perspectives — from genetics and epigenetics to genomic instability and tumor heterogeneity — by Schneider & Kulesz-Martin (89), Sigston & Williams (90), and Uthamacumaran & Zenil (15), pointing to the need for mathematical models and simulations to capture the complexity of cancer.

Parallelly, other innovations are highlighted by Thiong’o & Rutka (91) who discuss the potential of digital twin technology in customizing the treatment of pediatric cancers, while Faramarzpour et al. (92) and Hameroff (93) explore how the principles of quantum mechanics can revolutionize the understanding and therapeutic approach to cancer. Quantum theory, with its implications in quantum entanglement and coherence, offers new perspectives for intracellular communication and the development of less invasive and more effective therapies.

In complement to what was stated by Faramarzpour et al. (92) and Hameroff (93), Jerman et al. (94), and Kaluthanthri et al. (95) add that the importance of molecular dynamics coherence and quantum biology as organizing principles behind the coordination of biological processes can structure the new systemic vision of cancer development, as mechanisms associated with an active role in mediating the flow of energy and information in molecular systems.

Furthermore, Hollar (96) discusses the competition of ecological resonances in the quantum metabolic model of cancer, positioning the tumor as an ecological problem of quantum information, emphasizing the possibility of managing it through quantum information bit quantum (qubit) phase transitions and principles of thermodynamic hysteresis. However, there is also a critical issue being a unique paradigm regarding this complex physiopathological condition, being the ability of biostructures to amplify weak electromagnetic triggers, which provides a basis for transposing cellular destinies that can perpetuate cancer as a disorder (97).

By applying such quantum theoretical approaches, new dynamic models become accessible for evaluating more integrated stages of carcinogenesis, such as cell migration relations, paving advances on nanofabrication and image analysis, detailing cellular motility, and conceptualizing the emergent behavior of cells (98). In consonance, new models also leverage challenging visions like ecological resilience on cancer, which is based on the analysis of interactions between cancerous and normal cells. This suggests a therapeutic approach to the tumor as guiding the system to the attraction basin of a “healing” state, derived from resilience analysis for personalized oncological treatments (99).

As we proceed through modeling and structuring of potential laboratory studies based on initial computational results, chaos theory highlights a new theoretical perspective to explore the complex and often unpredictable dynamics of cancer, being capable of pointing towards possible research directions in radiotherapy, for example (100). But also, when connecting with quantum mechanics and new hypothesis, better descriptions regarding how cells can become cancerous through an increase in entropy, open doors to explore the understanding of tumor evolution and its biological, chemical, physical, and mathematical peculiarities, which are additionally referred to in **Table 1**.

**Table 1:** Some general highlights regarding the applications of chemistry, physics, mathematics, and biology in cancer study.

Description	Authors
Highlighted the complexity of chemotherapy resistance in cancer treatment, underlining the need for multidisciplinary approaches to develop more effective therapies.	Abdelmaksoud et al. (101)
Developed a “digital twin” of cancer,using AI to detect metastases in radiological reports, promising advances in personalized medicine.	Batch et al. (102)
Explored the complexity of cellulardifferentiation and the development of an epigenetic landscape, illustrating the flexibility of cell fate and the importance of models to predict specific cellular outcomes.	Bhattacharya, Zhang and Andersen (103)
Highlighted the importance of genetic regulatory networks in development and evolution, evidencing self-organization as fundamental in the formation of complex structures.	Bozorgmehr (104)
Approached cancer as an atavistic condition, proposing treatment strategies that exploit the predictability of cancer’s genetic “toolkit” for personalized therapies.	Davies & Lineweaver (105) and Greaves (106)
Explored physical and quantum approaches to understand cell migration and cancer, suggesting more integrated models to explain complex biological processes.	Brückner & Broedersz (98), Demetrius et al. (107), Bordonaro & Ogryzko (108), and Djordjevic & Djordjevic (109)
Discussed the reconciliation between theories of carcinogenesis through systems biology, suggesting that cancer exists in states of self-organized criticality.	Grunt & Heller (110)
Proposed a connection between tissue specialization in the evolution of multicellularity and cancer development, highlighting phenotypic plasticity as a crucial factor.	Hammarlund et al. (111)
Addressed the importance of mechanical and chemical signals in cell biology, focusing on EMT and vascular adaptation, respectively.	Humphrey (112) and Tripathi, Levine & Jolly (113)
Explored *Dictyostelium discoideum* as a model to understand cellular cooperation and competition, with implications for cancer research.	Kawli & Kaushik (114)
Highlighted the importance of key proteins in the response to replication stress and cell cycle control in cancer, suggesting quantum biology to find new therapies.	Khamidullina et al. (115)
Investigated the correlation between nuclear morphology and the survival of cells treated with cisplatin, emphasizing multinucleated polyploidy and chemotherapy resistance.	Kim et al. (116)
Analyzed cancer metastasis, emphasizing the tumor microenvironment, phenotypic heterogeneity, cellular plasticity, and cell mechanics as crucial factors for progression.	Mierke (117)
Emphasized the adaptive response of cells to anticancer treatment and natural selection, showing the importance of physical and biological modifications in cancer resistance.	Mittal et al. (118) and Jacobeen et al. (119)
Discussed the loss of coherence as contributing to cancer development, suggesting the restoration of coherence as a therapeutic strategy.	Plankar & Jerman (120)
Applied chaos theory and fractal mathematics to the study of cancer, focusing on metabolism and the immune system as targets for treatment.	Sharma (121)
Discussed the importance of cellular mechanical memory and physical principles in tissue organization, with implications for cancer.	Trepat & Sahai (122) and Price et al. (123)

**Figure 3:**
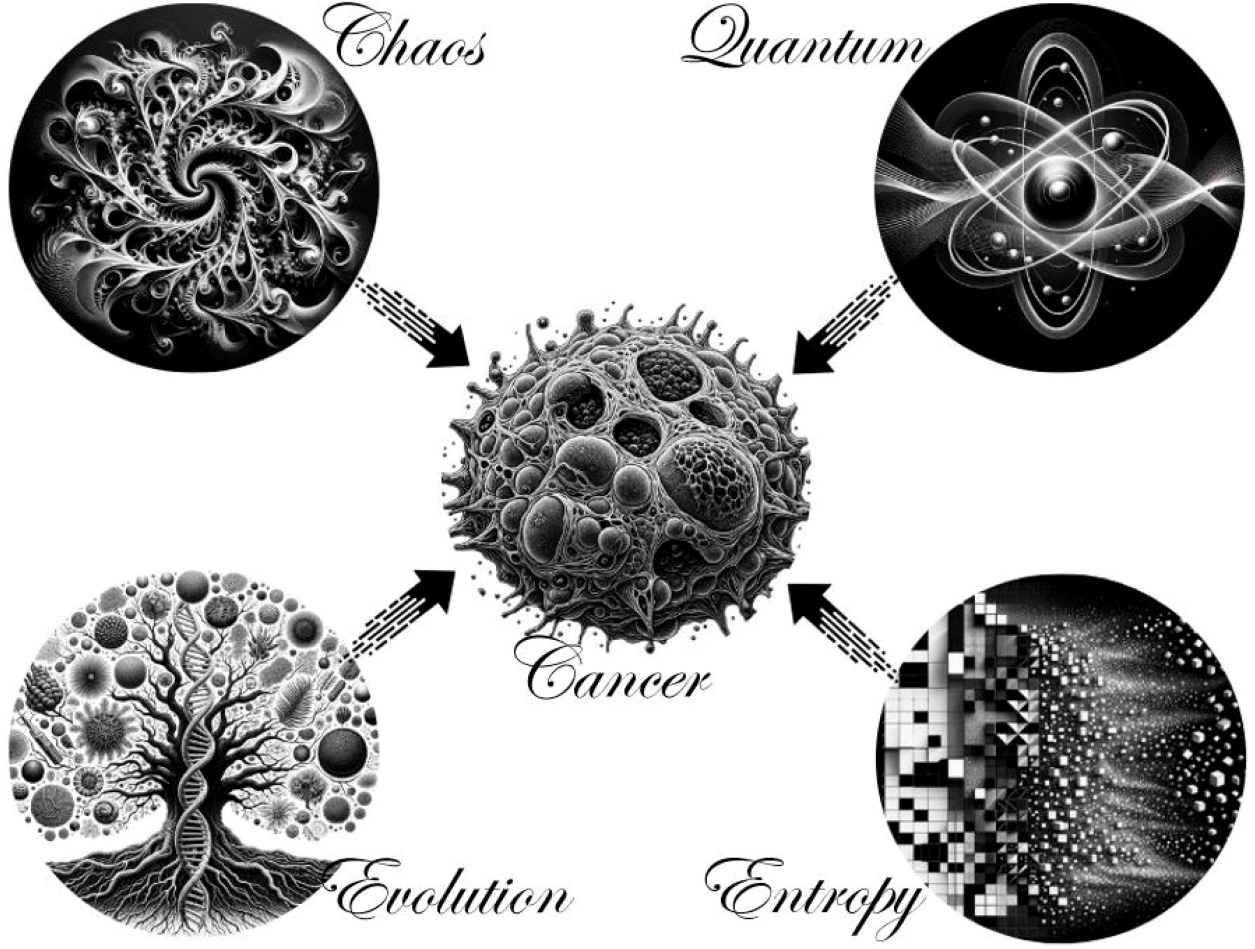
A perspective of new scientific directions in oncological research. Chaos theory incorporates unpredictability and dynamism according to fractal patterns like the design of the Lorenz attractor. In addition, quantum supplements atomic evaluations and singular details regarding the behavior of particles in cancer as represented in stylized atoms and probabilistic waves. At the same time with entropy, the tumor heads towards a constant progression of molecular, structural, cellular, and systemic changes that move from order to a chaotic landscape with the influence of time on an increasing disorder. Finally, there is a “mechanistic bridge” provided by evolution combined with genetics, because of the relationships of genomic instability and rearrangements, cellular diversity/heterogeneity, and systemic connection in the immense tumor mass in constant evolution. Note: This image was created with the assistance of AI and Canva.

Thus, despite so many novelties, the importance of human and molecular genetics viewed from a multidisciplinary perspective is also observed, according to Ferreira et al. (124), highlighting the relevance of epigenetics, genetic testing, and genetic counseling in the prevention, early diagnosis, and treatment of various tumors. But also, according to Casotti et al. (125–128), a union with bioinformatics as an essential tool in oncogenetics achieves deeper and more accurate analyses in complex cancer cases, through the application of software and platforms as well as methodologies of systems biology and single-cell biology (125–128). Chaos theory, highlighting unpredictability and patterns like the Lorenz attractor, along with quantum theory’s focus on atomic and subatomic behavior, contribute to understanding cancer’s complexity. These theories illustrate how cancer evolves from order to chaos through continuous molecular, structural, and cellular changes, driven by entropy and time. Additionally, evolutionary and genetic mechanisms act as a “mechanistic bridge”, facilitating the understanding of cancer’s progression, genomic instability, cell diversity, and the systemic nature of tumor growth, thus, there is a deep connection between evolutionary and genetic bases along with understandings at atomic levels provided by chaos theory, quantum, and entropy, as outlined in **Figure 3**.

**Figure 4:**
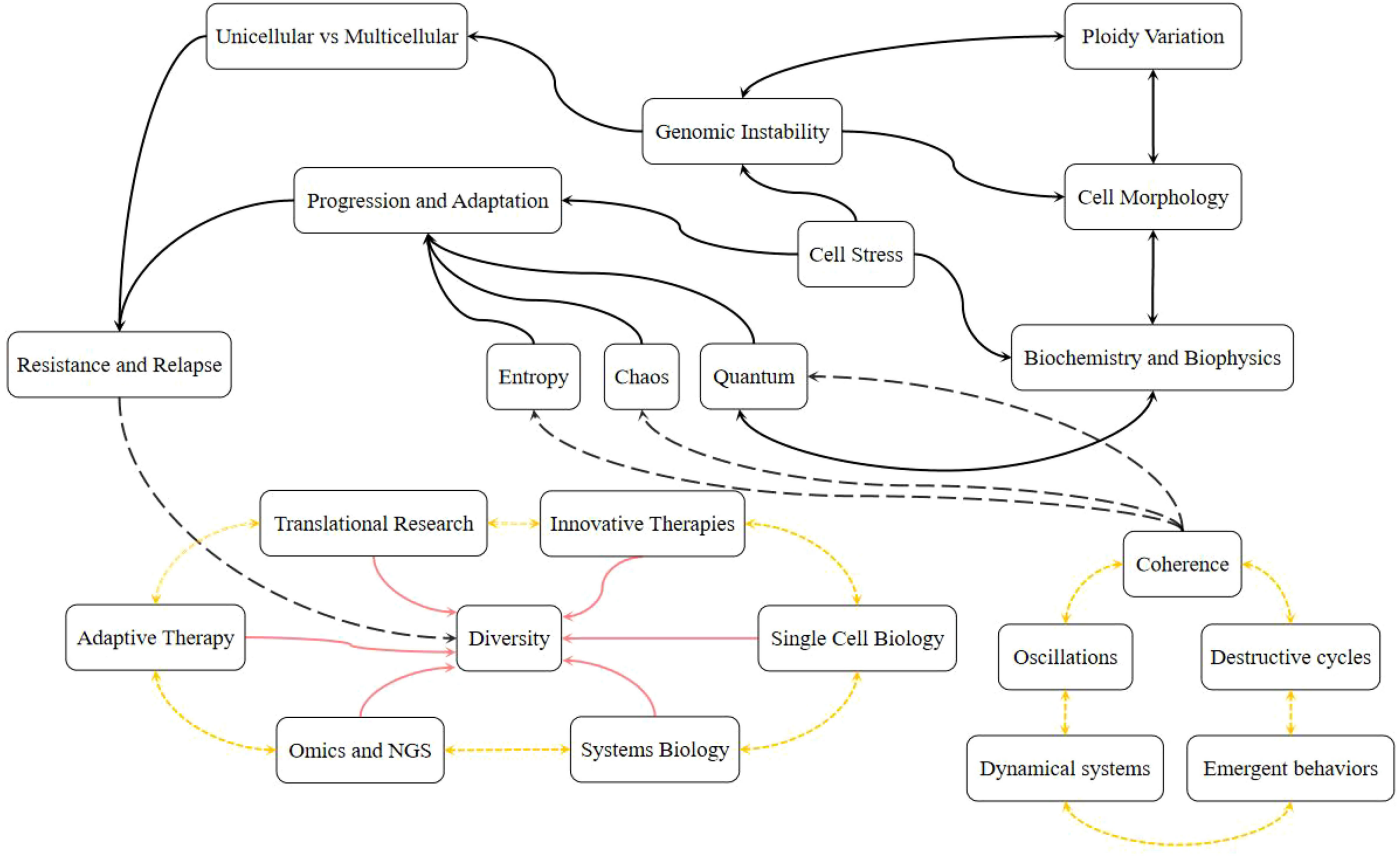
Innovative perspectives on cancer evolution and adaptation & advanced methodological approaches to stressful cellular events. Schematic compilation regarding the concepts discussed throughout the article and potential directions concerning innovative and pioneering methodological approaches applicable to the elucidation and interpretation of the evolutionary progression and adaptation of cancer in the face of the cell’s stressful occurrences.

Amidst such highlights, new initiatives based on single-cell biology, holistics, quantum and evolution become valuable areas for a more detailed elucidation with prominent clinical application through more personalized methodologies and based on adaptive therapy emphasizing the real ability to modulate or control or tame cancer. Therefore, concepts about stress and chaos need to merge with translational approaches across multiple areas of knowledge such as medicine, pharmacy, biology, physics, chemistry, and others, with a common goal, scientific diversity can translate into new effective achievements in oncological treatment and tumor biology, as outlined in **Figure 4** which emphasizes the concepts presented throughout this article and probable methodological directions for laboratory studies.

#### 3.4.1 Current technologies

The new technologies and techniques being incorporated into the study of cancer biology stem from increasing interaction between systems biology, evolutionary biology, quantum biology, and single-cell biology. This allows for a more comprehensive understanding of cancer, from identifying new cellular subtypes to understanding mechanisms of drug resistance. In this regard, numerous researchers are investing valuable efforts in increasingly collaborative and integrated research areas.

In parallel, the reconstruction of tumor trajectories using imaging has been complemented by detailed single-cell profiling with multi-omic characterizations, deep phenotyping, and real-time monitoring of evolution and treatment response. This allows for adjustments in therapies according to changes detected in the tumor microenvironment and cell populations (longitudinal, multiscale, and translational monitoring). Furthermore, the use of sustainable bioproducts for developing new, less toxic, and more effective therapies is also being explored.

##### 3.4.1.1 Modelling, imaging, computing, and systems biology

Studies by Rockne et al. (129) highlighted the importance of mathematical oncology, with mathematical models for personalizing cancer treatments. These models enable the creation of individualized screening strategies, therapeutic response predictions, and adaptive treatment plans. Additionally, mathematical models can incorporate various imaging techniques and tumor measurements. Thus, tumor trajectories can be reconstructed during therapy through the integration of multiple measurement modalities, providing precise determination of tumor size changes during treatment and evaluating the response or progression of the disease. This is crucial for implementing therapeutic strategies based on systems biology and evolutionary biology (130).

Systems biology, in combination with modeling, also advocates for the introduction of new software platforms for the management, integration, and analysis of oncological data, such as the Digital Slide Archive (DSA), which encompasses large collections of histological images, integrating them with clinical and genomic metadata. Consequently, digitized histological slides provide high-resolution images that capture cytological and microenvironmental details of tumor tissue, facilitating the visualization and quantitative analysis of these images (131). Given the advancements in imaging, new frontiers in image quantification become accessible to the evolutionary and ecological scope of cancer, emphasizing tumor heterogeneity and the evolutionary dynamics of malignant cells. This is achieved through quantitative image analysis with new machine learning and artificial intelligence techniques, enabling the extraction of mineable data that can be correlated with molecular and cellular properties of tumors. Therefore, a theoretical framework is offered to predict emerging phenotypes, aiding in the development of evolution-based therapies (132).

In line with image quantification, computational efforts have advanced cancer research by including computational biology and artificial intelligence (AI) technology. This is justified as omics and molecular advances have presented unprecedented opportunities, but also require new approaches to capture, organize, and analyze large-scale heterogeneous data. Thus, the combination of experimental and computational methods has embraced comprehensive, integrated, and translational understandings in elucidating tumor biology, leading to more accurate diagnoses and more effective therapies (133). In view of this, AI has been enhancing FDA-approved medical devices capable of extracting relevant information from vast medical data sets, facilitating more precise diagnoses and personalized treatments, and in the process of new drug discovery, through the precision of models and integration of complex data (134).

##### 3.4.1.2 Multi-omics and single-cell

The evolution of understanding about tumor biology has significantly benefited from advances in sequencing and data analysis technologies, especially in the context of intratumoral heterogeneity and cancer evolution dynamics. Several studies contribute to forming a detailed and integrated view of this complexity, combining single-cell approaches with advanced computational strategies and integration of multi-omic data.

Aparicio et al. (135) expanded the analysis of tumor evolution by utilizing single-cell genomic sequencing. They combined mathematical and computational methods to analyze topological data, creating simplified skeletal graphs representing high-dimensional data geometry. This approach revealed the complexity and variation among cancerous and healthy cells, offering a granular view of cellular heterogeneity in tumors, crucial for understanding cancer progression and adaptation at the cellular level.

Complementing this view, Stransky et al. (136) highlighted the integration of “omic” information into dynamic models using both “bottom-up” and “top-down” strategies. The “bottom-up” strategy derives experimental information about the structure and relationships between biological system components, while the “top-down” approach applies theoretical knowledge to model system mechanisms and dynamics. The combination of these strategies allowed for the creation of multi-scale models covering mechanistic details and a broad view of biology, essential for understanding the complexity of cancer systems.

Nam et al. (137) emphasized the importance of integrating genetic and non-genetic determinants in cancer evolution using emerging single-cell multi-omics technologies. These technologies captured and integrated multiple data modalities, analyzing cellular states, epigenetic profiles, spatial distributions, and interactions with the tumor microenvironment. This detailed analysis revealed intratumoral diversity as a crucial factor in progression, relapse, and treatment resistance.

To address the integration of these complex data, Adossa et al. (138) added computational strategies for integrating single-cell multi-omic data. They categorized integration approaches as early, intermediate, and late. Intermediate integration, for example, used joint dimensionality reduction and statistical modeling, allowing integrated analysis of different omic layers. This provided a deeper understanding of cellular heterogeneity in tumors and helped identify cell differentiation trajectories.

Delving into the potential of single-cell RNA sequencing to explore tumor heterogeneity at spatial levels, Ahmed et al. (139) discussed the benefits associated with techniques like Drop-Seq and SCRB-Seq, which allowed profiling of the transcriptome of individual cells, revealing the complexity of cell composition in tumors. Advances in spatial sequencing, such as MERFISH and FISSEQ, map gene expression to the physical location of cancerous tissues, providing insights into the spatial organization of tumor cells.

Furthermore, Casado-Vela et al. (140) discuss the importance of integrating omics approaches with advances in mass spectrometry and other high-precision technologies, which have allowed identification and quantification of biomolecules on a large scale, providing fundamental data for cancer research. Simultaneously, regarding advances in single-cell expression profiling, Gonzalez Castro et al. (141) highlighted the valuable benefits from single-cell genomics and transcriptomics techniques, revealing intratumoral heterogeneity (ITH), identifying cell subpopulations influencing cancer progression and drug resistance. These advanced methods have enabled the analysis of multiple tumor aspects, including genome, transcriptome, epigenome, and proteome, providing deep insights into tumor evolution, interactions with the tumor microenvironment, and therapeutic response.

##### 3.4.1.3 Innovations

Beyond the aforementioned advancements, new breakthroughs are being made in the interplay between engineering, holistic approaches, laboratory practice, and cancer. Weltin et al. (142) introduced an innovative multiparametric microphysiometric system to dynamically monitor the metabolism of human cancer cells. This system integrates chemical sensors and biosensors on a glass microchip, enabling precise measurements of pH, oxygen, glucose, and lactate in cell cultures. The use of such microfluidics provides detailed control and continuous analysis of cellular metabolism, offering valuable insights into the response of cancer cells to different treatments and the evolution of their metabolism.

Concurrently, Lovitt et al. (143) highlighted the importance of three-dimensional (3D) cell culture techniques in cancer drug discovery. They argue that 3D cell cultures are more representative of the *in vivo* tumor microenvironment compared to two-dimensional (2D) cultures, allowing for a more realistic investigation of mechanical processes and drug resistance. The adoption of 3D models in drug discovery programs is becoming more common, with methods including non-adherent cell cultures (anchorage-independent) and 3D structures that adhere to a substrate (anchorage-dependent), essential for better understanding the mechanisms of drug action and their efficacy.

The issue of drug resistance in cancer treatment is addressed by Craig et al. (144), who highlight how medical engineering, including nanotechnology and computational modeling, is being used to tackle this challenge. Nanotechnology has revolutionized drug delivery, allowing for the selective destruction of tumor cells and overcoming failures in immunotherapies. Bioengineered tumor models create physiologically relevant environments to predict clinical refractoriness and test drug combinations, personalizing treatment for each patient.

Furthermore, Gao et al. (145) emphasize the crucial role of holistic approaches in optimizing biotechnological processes and improving the efficiency of extracting and transforming biological materials into high-value products. In the oncological context, these technologies are applied to develop new biomarkers and therapeutic agents from renewable sources, contributing to more sustainable and personalized treatments.

The integration of systems biology, evolutionary biology, quantum biology, and single-cell biology is creating new frontiers in cancer research. The technologies and techniques described by the authors provide a robust foundation for tackling the complexity of cancer, offering hope for the development of more effective and personalized therapies. The combination of these disciplines allows for a deeper and more detailed understanding of tumor complexity, enabling the development of innovative and personalized therapeutic strategies. The ability to monitor and respond to changes in the tumor at the cellular and molecular levels promises to significantly improve outcomes for cancer patients, marking a new era in precision medicine. In summary, the convergence of these technologies and techniques in the various aforementioned fields foresees new interdisciplinary, collaborative, and integrated pathways.

#### 3.4.2 Challenges and limitations

The integration of different fields in cancer research faces numerous challenges and limitations, such as understanding genomic heterogeneity and the continuous evolution of tumors, the complexity of polyploidy in organisms, and the dynamics of chromosomal rearrangements in cancer immortality, among other highlights. Thus, these highlights demand multidisciplinary approaches and advanced analytical methods for a more comprehensive understanding of cancer biology, essential for developing more effective and personalized therapeutic strategies. Studies indicate the need for an integrated approach that considers evolutionary complexity, tumor heterogeneity, and the temporal order of biological events in cancer development. To overcome these challenges, a combination of basic research, the development of new technologies, and careful clinical application is necessary to translate advances in cancer biology into better patient outcomes. This is well represented by the works of Anand et al. (146), Blischak et al. (147), Duesberg & McCormack (148), Enriquez-Navas et al. (149), Gallaher et al. (150), and Gourmet et al. (151).

This is because researchers like Anand et al. (146), focusing on the genomic heterogeneity and continuous evolution of malignant brain tumors, such as glioblastomas, have highlighted the importance of multi-omics profiles to improve personalized therapeutic strategies. Blischak et al. (147) addressed the complexity of polyploidy in organisms, discussing the influence of the environment on the selection of ploidy levels in fungi and phylogenetically shared in tumors. Duesberg & McCormack (148) proposed a chromosomal theory of immortality in cancers, suggesting that clonal and flexible chromosomal rearrangements generate cancer immortality, and developing techniques targeted at these rearrangements is immensely useful but very challenging.

In parallel, Enriquez-Navas et al. (149), Gallaher et al. (150), and Gourmet et al. (151) highlight the obstacles faced when trying to incorporate evolutionary and systems approaches into cancer therapy. Enriquez-Navas et al. argue that cancers develop resistance immediately after the application of any therapy, suggesting the need for adaptive strategies. Gallaher et al. emphasize the importance of precise measurement of tumor burden over time for effective therapeutic decisions. Gourmet et al. identify a temporal order of cancer hallmarks, suggesting that genomic instability is the first hallmark and immune evasion the last, highlighting the complexity of translating bioinformatics discoveries into clinical practice.

Supplementing the above, the different aspects of cancer biology represent a challenge for creating targeted clinical interventions. Thus, the integration of external data with phylogenetic trees provides a valuable source of information, but complex due to the diversity of formats and external data, presenting barriers to visualization given the complexity of multidimensional information. Moreover, high-quality data sets and technical challenges for temporal analysis and understanding chaotic systems add to existing obstacles.

However, despite the present difficulties, numerous researchers have achieved prominent accomplishments, such as Hardie et al. (152) discussing genome size variation and measurement technique limitations; Huang et al. (153) with solutions to the complexity of regulatory networks in tumorigenesis; Jiao et al. (43) with hypotheses on the role of dormant cancer cells, especially PGCCs; Kirsch-Volders et al. (154) exploring the role of tetraploidy in cancer development and the challenges in understanding the relationship between tetraploidization and carcinogenesis; Li et al. (155) advancing in technical challenges and eliminating artifacts brought by single-cell sequencing technologies; Lidke et al. (156), Jana et al. (157), and Mirzayans (158) emphasizing the importance of the tumor microenvironment in modulating dysfunctional cellular processes that drive malignant transformation and treatment resistance, highlighting the need for more sophisticated therapeutic approaches.

Beyond highlights associated with deeply biological issues, challenges and limitations are present in connecting biology with profound mathematical, computational, and physical content. This is due to the negative preconception among biologists towards the involvement of calculations. However, works like Pell et al. (159) highlight the potential use of computational areas by digital pathology technologies to improve tumor evaluation and treatment response; attractor landscape analyses, as proposed by Shin and Cho (160), and the educational strategies suggested by Škarková et al. (161), aimed at re-educating cancer cells, face significant challenges in practical implementation from experimental perspectives, but also regarding mathematical modeling, as explicitly stated by West et al. (84) in exploring adaptive therapy through mathematical modeling and verifying the need for rigorous clinical validation.

Furthermore, Uthamacumaran & Zenil (15) and Uthamacumaran (162) highlight the importance of understanding the physics that permeates the dynamic systems of cancer, emphasizing the need for detailed temporal data and causal inference methods, exacerbating statistical challenges. Adding to the physical aspect, understanding the biophysical properties of cancer cells and the tumor microenvironment can offer new insights into identifying effective therapeutic targets and improving treatment evaluation and follow-up, as highlighted by Xuan et al. (163), but at the same time accompanied by conceptual and technical challenges. In new technological pathways, Uthamacumaran (46) proposes the potential of combining single-cell technologies and computational models to provide deeper hypotheses and ideas about cellular cybernetics and the prediction of cellular fates in precision oncology. However, these advances depend on greater collaboration between disciplines, the development of new technologies, and the adaptation of concepts from complex systems and cellular cybernetics to understand and treat cancer more effectively.

**Figure 5:**
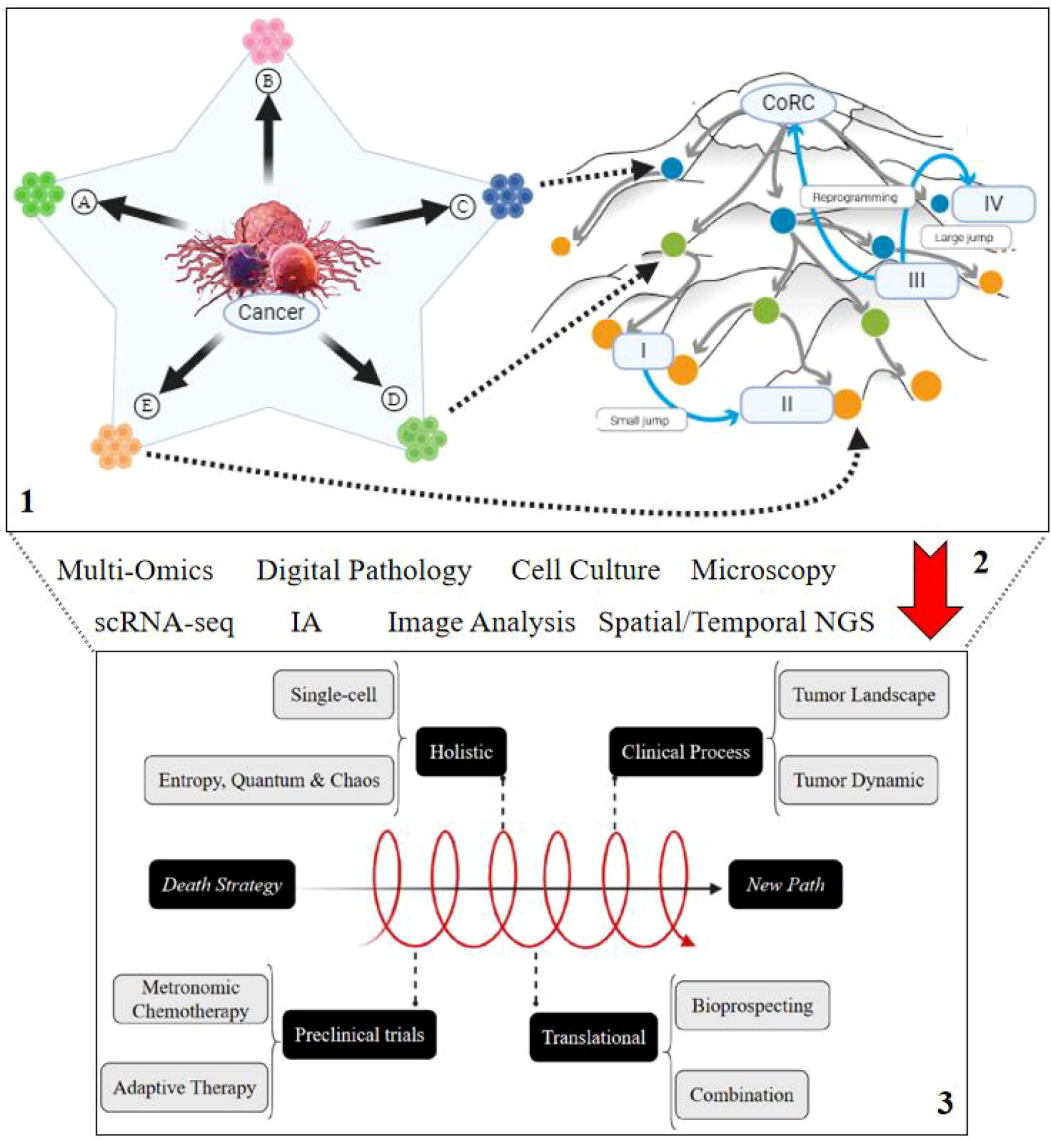
Integration generating innovation. 1. Throughout the article, we aimed to highlight the immense potential of connecting the physical, chemical, and biological study of the tumor cell to elucidate the phenotypic diversity varying among multiple onco-attractor microstates **(A–E)**, which can comprise different moments in an epigenetic, genetic, and quantum landscape. It is possible to shape phenotypic characteristics through “jumps” along each attractor state (I to II or III to IV) due to the plasticity and reprogramming of self-organized cancer cells. 2. Given the challenging complexity of understanding so much information mixed in a heterogeneous tumor mass, new technological advancements will be required. These include multi-omic approaches, 3D cell cultures, advanced microscopy methodologies linked to automated image analyses and AI, fourth-generation and single-cell sequencing, digital pathology, and numerous other techniques or methodologies to elucidate and connect basic and clinical research. 3. Consequently, the structural paradigms of the “death strategy,” which drive a more aggressive progression of cancer accompanied by intense side effects in patients, will be reshaped. As new breakthroughs are achieved through holistic and singular research, containing preclinical trials representative of the real evolutionary progression of the tumor through adapted methodologies, the gap in translational research will be bridged. This will pave the way for new drug combinations along with the bioeconomic and bioprospecting of an extensive source of antineoplastic compounds still unknown in marine and terrestrial biodiversity. There will be a new direction for clinical research that integrates the landscape and dynamics of the tumor.

In this aspect, although significant advances have been made, the effective integration of various disciplines in oncology requires a collaborative approach, overcoming technical, computational, and conceptual challenges. Thus, the union of efforts can transform cancer into a manageable chronic disease, prolonging patient survival and improving their quality of life. Therefore, more studies and collaborations are needed to overcome the challenges and limits and translate these promises into tangible clinical benefits, as compiled in **Figure 5** below.

## 4 Conclusion

This article aimed to explore the complex intersection of evolutionary biology, single-cell biology, systems biology, comparative oncology, translational research, quantum biology, and chaos theory in cancer research, while also paving new avenues for enhanced flexibility in the increasingly multidisciplinary, cooperative, and open field of oncology research. It emphasized the critical need for integrating knowledge from biology, physics, chemistry, informatics, and engineering, highlighting the potential for significant advancements that could transform personalized and adaptive cancer therapies. This integration is poised to forge new healthcare models aligning with One Health and sustainable development principles, design adaptable clinical trials with real-time monitoring, discover novel compounds and biomarkers via single-cell analysis, consider bioeconomic factors, minimize treatment side effects, and improve patient quality of life. Single-cell biology has sparked a revolution in grasping the diversity of cancer, enabling the creation of more precise and personalized treatments. Adaptive and translational research has further sped up the translation of scientific breakthroughs to clinical applications, swiftly adapting to the unique requirements of each patient and the dynamic nature of cancer. The merger of systems biology with quantum physics is opening up innovative ways to understand cancer’s intricacies, though the application of these insights to develop concrete treatments remains in the nascent stages.
